# Supplementary material for: Nanoalignment by critical Casimir torques
Source: Nat Commun. 2024 Jun 14;15:5086. doi: 10.1038/s41467-024-49220-1 (PMC11178905; doi:10.1038/s41467-024-49220-1)
Supplement: Supplementary file 1 — Supplementary Info [file 41467_2024_49220_MOESM1_ESM.pdf]

Supplementary material to "Nanoalignment by Critical Casimir Torques"

Gan Wang,[1,*] Piotr Nowakowski,[2,3,4,*] Nima Farahmand Bafi,[2,3,5] Benjamin Midtvedt,[1] Falko Schmidt,[6] Agnese Callegari,[1] Ruggero Verre,[7] Mikael Käll,[7] S. Dietrich,[2,3] Svyatoslav Kondrat,[2,3,5,8,†] and Giovanni Volpe[1,‡]

[1]*Department of Physics, University of Gothenburg, SE-41296, Gothenburg, Sweden*

[2]*Max Planck Institute for Intelligent Systems,*
Heisenbergstraße 3, D-70569 Stuttgart, Germany

[3]*IVth Institute for Theoretical Physics, University of Stuttgart,*
Pfaffenwaldring 57, D-70569 Stuttgart, Germany

[4]*Group of Computational Life Sciences, Division of Physical Chemistry,*
Ruđer Bošković Institute, Bijenička cesta 54, 10000 Zagreb, Croatia

[5]*Institute of Physical Chemistry, Polish Academy of Sciences, 01-224 Warsaw, Poland*

[6]*Nanophotonic Systems Laboratory, Department of Mechanical and Process Enginnering,*
ETH Zürich, CH-8092, Zürich, Switzerland

[7]*Department of Physics, Chalmers University of Technology, SE-41296, Gothenburg, Sweden*

[8]*Institute for Computational Physics, University of Stuttgart,*
Allmandring 3, D-70569, Stuttgart, Germany

[*] These authors contributed equally to this study.

[†] skondrat@ichf.edu.pl; svyatoslav.kondrat@gmail.com

[‡] giovanni.volpe@physics.gu.se

SUPPLEMENTARY FIGURES

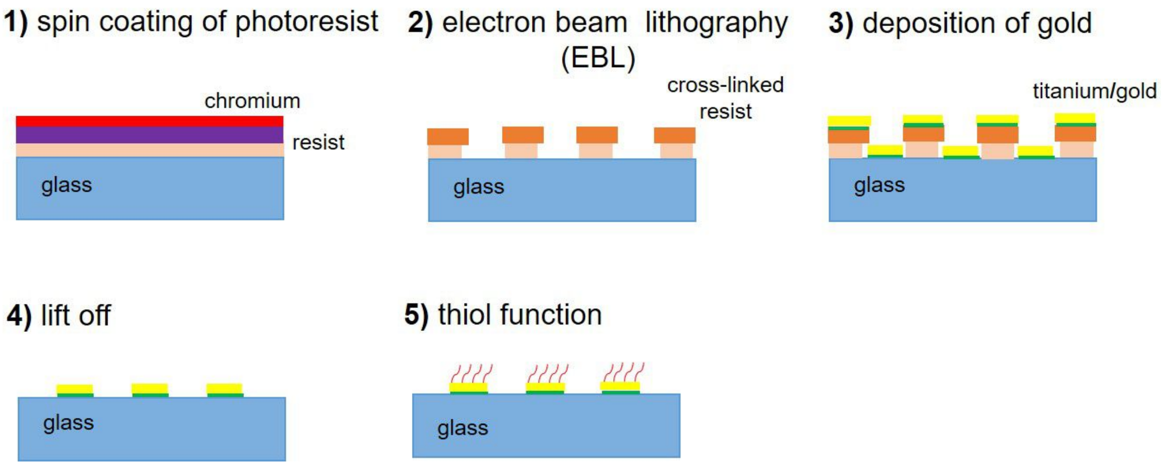

Figure S1. **Sketch of the steps used to fabricate the patches on a glass substrate.** Illustration of the key fabrication steps: (**1**) Spin coat a double-layer resist (LOR 3A (pale pink)/UVN 2300 (purple)) on glass: LOR 3A acts as the sacrificial release layer for the lift-off process, while UVN 2300 serves as the top negative photoresist. Deposition of a 25 nm thick chromium film as a conductive layer for subsequent electron beam lithography (EBL) steps. (**2**) Definition of the pattern features in the resist by EBL. The chromium layer is chemically etched after exposure to the EBL. The orange regions represent the cross-linked photoresist which becomes less soluble due to the exposure to the electron beam, making them resistant to development, while the developer solvent (MF-CD26) removes the unexposed areas, which are the empty regions in the image. (**3**) Deposition of 2 nm titanium (green parts) and 25 nm gold (yellow parts) on top of the cross-linked photoresist and the glass substrate. Titanium is used as an adhesion layer between glass and gold. (**4**) Formation of the patterns by lifting off the deposited titanium and gold. The samples are soaked in an organic solvent for 2 hours with the aim of removing the photoresist and the metal above that while the metal on the substrate remains unaffected. (**5**) Functionalization of the gold surface with octanel thiols makes it hydrophobic. The remaining glass surface is hydrophilic.

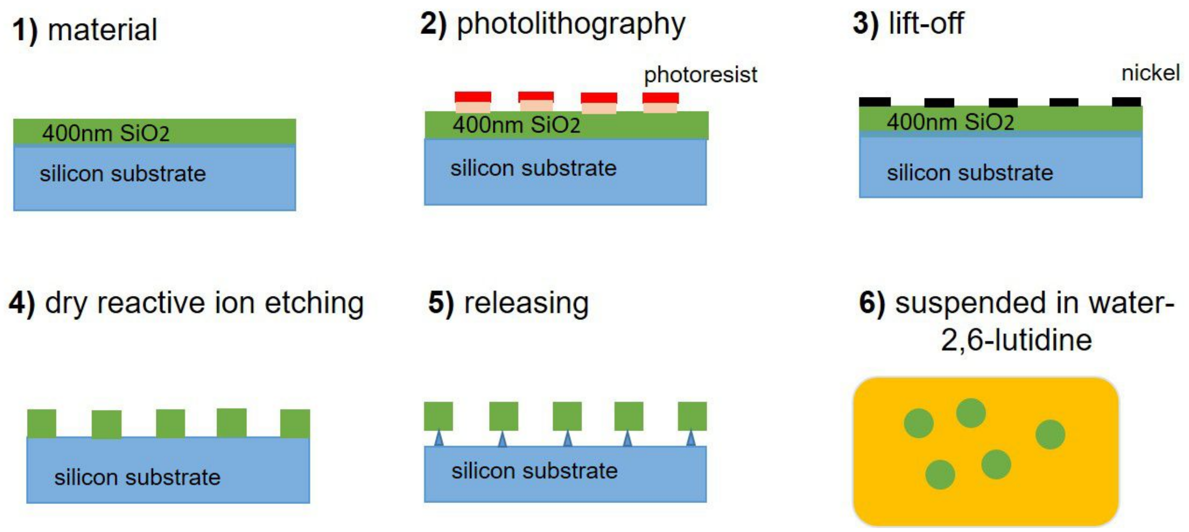

Figure S2. **Sketch of the steps used to fabricate the microdisks.** Illustration of the key fabrication steps: (**1**) Initial substrate consisting of a standard 4-inch silicon wafer with a 400 nm-thick SiO_2. (**2**) Definition of the features of the microdisks in a double layer resist (LOR 3A (pale pink)/S1805 (red), LOR 3A acts as the sacrificial release layer for the lift-off process, while S1805 serves as the top positive photoresist) by using a Heidelberg DWL2000 direct laser writing. (**3**) The creation of a 40 nm nickel hard mask involves the use of a lift-off process, wherein the photoresist is removed by soaking it in hot Remover 1165 for a duration of 2 hours. (**4**) The formation of the structures of the microdisks uses dry reactive ion etching with CHF_3. The nickel layer is chemically etched after the dry reactive ion etching process. (**5**) Release of microdisks from the silicon wafer by using isotropic reactive ion etching with SF_6. With this, the microdisk has only small contact points with the substrate, facilitating subsequent particle release into the solution via sonication. (**6**) Suspension of microdisks in a critical mixture of water and 2,6-lutidine obtained by sonication.

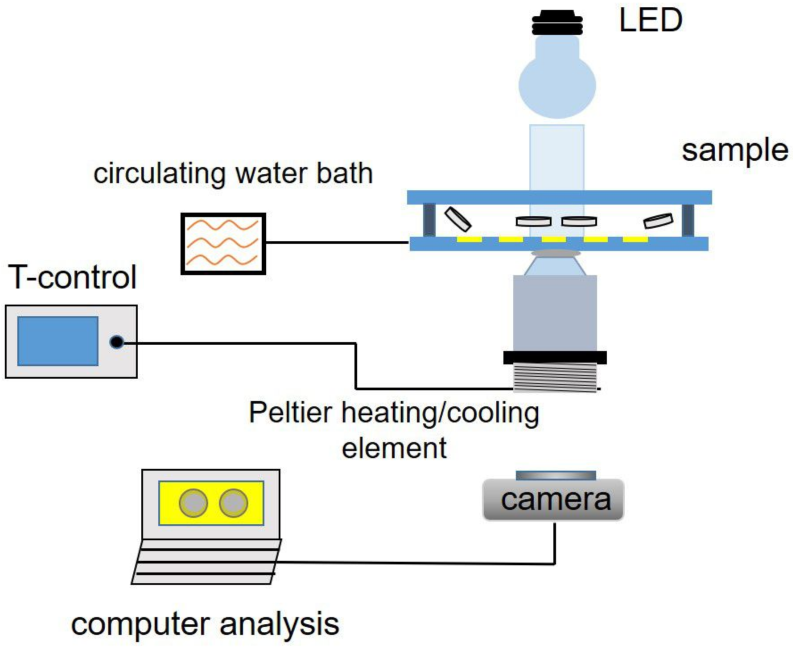

Figure S3. **Scheme of the experimental set-up for temperature control of the sample.** The home-made set-up combines digital video microscopy and temperature control elements. The digital video microscopy consists of a white light (LED) illumination and a 100 $\times$ objective to collect the scattered light; the camera is connected to a computer in order to acquire videos and then analyze the movement of the particles. The temperature control system is divided into two parts: first, a circulating water bath is used for a rough temperature control of the sample ($\Delta T = \pm 100\,\mathrm{mK}$); second, a Peltier heating/cooling element is mounted on the oil-immersion objective and precisely controls the temperature of sample ($\Delta T = \pm 10\,\mathrm{mK}$). In this way, the temperature of the sample can be reliably stabilized to better than $\pm 20\,\mathrm{mK}$.

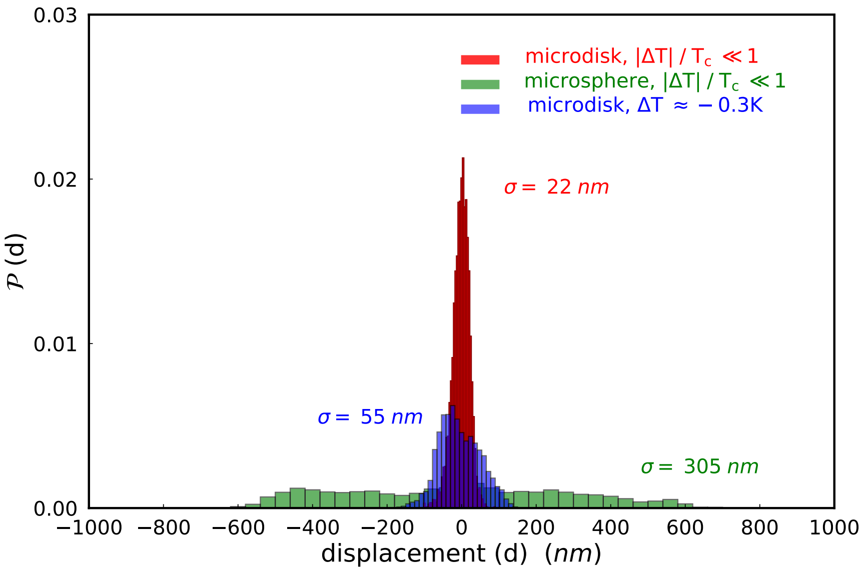

Figure S4. **Localization of a microdisk and a microsphere above a circular micropattern.** The experimentally determined displacement distribution along the x-axis of a microdisk at $|\Delta T|/T_{\mathrm{c}} \ll 1$ (red histogram), which is narrower than at $\Delta T = T - T_{\mathrm{c}} \approx -0.3\,\mathrm{K}$ (blue histogram), indicates strong confinement if the temperature is close to the critical temperature. Nevertheless, the distribution is broadest for a microsphere trapped at $|\Delta T|/T_{\mathrm{c}} \ll 1$ (green histogram) due to the smaller effective interaction area. The displacement standard deviations σ for the three conditions are 22 nm (red, microdisk at $|\Delta T|/T_{\mathrm{c}} \ll 1$), 55 nm (blue, microdisk at $\Delta T \approx -0.3\,\mathrm{K}$), and 305 nm (green, microsphere at $|\Delta T|/T_{\mathrm{c}} \ll 1$). Source data are provided as a Source Data file.

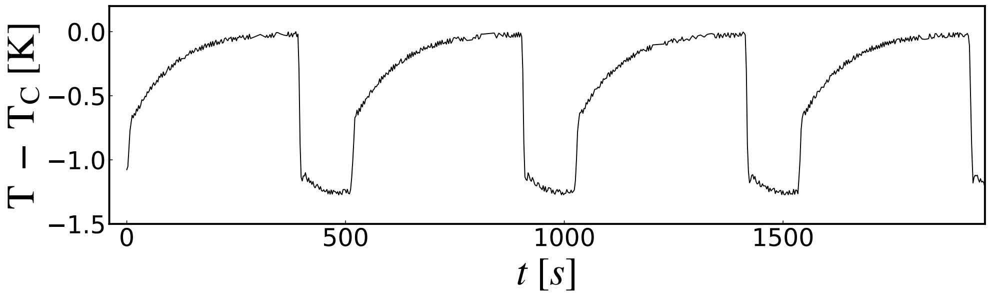

Figure S5. **Temperature control cycle of the sample.** Periodic raising and lowering of the temperature T towards and away from the critical temperature T_c in order to modulate the translational motion of microdisks between states of free diffusion and directed motion on a patterned substrate. This strategy is employed in order to enable the controlled long-distance movement of the microdisks over a distance exceeding one hundred micrometers, relative to a microsdisk size of 2.4 µm. Source data are provided as a Source Data file.

S1. SUPPLEMENTARY METHODS

A. Particle detection and tracking

The positions and, where needed, the orientations of the particles were tracked by using one of four methods which are described in the following subsections, depending on the kind of particle and of its configuration.

1. Position and orientation of a perpendicularly trapped microdisk

The position of a microdisk perpendicularly trapped at a pattern was tracked using the threshold method [1]. The grayscale images acquired by the camera were thresholded to produce a binary segmentation, the largest connected area of which corresponds to the microdisk. Subsequently, its centroid was computed in order to determine the microdisk position. Furthermore, its major and minor axes were computed to determine the microdisk orientation.

2. Position of a horizontally trapped microdisk

The position of a microdisk horizontally trapped at a pattern was tracked with a neural network using the Python package DeepTrack 2 [2]. Specifically, we employed a convolutional neural network (CNN) [3]. The input of this neural network was an image of the microdisk, and its outputs were the x and y coordinates of the microdisk. The network was trained on synthetic data also generated with DeepTrack 2.

3. Position of a freely diffusing microdisk

The position of a freely diffusing microdisk was tracked using a self-supervised neural network named LodeSTAR [4]. The advantage of using this approach was that it could be trained on a few

(5) images of the freely diffusing microdisk without the need to know *a priori* its exact position.

4. Position and orientation of a chiral microparticle

The position and orientation of a chiral microparticle trapped at a pattern were tracked with an appositely developed method. First, in the initial frame, two corners of the particle (not overlapping with the pattern, because the overlapping ones could not be reliably identified) were manually identified and the remaining corners were determined taking into account the known shape and dimensions of the chiral microparticle. Then, in each subsequent frame, the particle corners were searched for in an area of radius 5 pixel from their previous location by thresholding the image [1]; again, only corners not overlapping with the pattern were retained in order to determine the particle configuration (again taking into account the known shape and dimensions of the chiral microparticle).

B. Interaction potential between a microdisk and a patterned substrate

We consider a disk-shaped microparticle of radius R and thickness W, the center of which is at a distance D_c above a patterned surface (see Fig. S6). The surface pattern consists of a circle of radius a with the same boundary condition as the microdisk and the opposite one for the remaining part of the surface. The particle can be shifted a distance s away from the center of the pattern along any arbitrary direction, which we assume to be the x-axis without loss of generality. We define the orientation of the microdisk by initially positioning it parallel to the surface; subsequently, we perform two rotations: the first rotation is by an angle α around an axis parallel to the x-axis passing through the center of the microdisk, and the second rotation is by an angle γ around an axis parallel to the y-axis also passing through the center of the microdisk. Thus, a set of four variables, D_c, s, α, and γ, unambiguously define the location and the orientation of the microdisk,

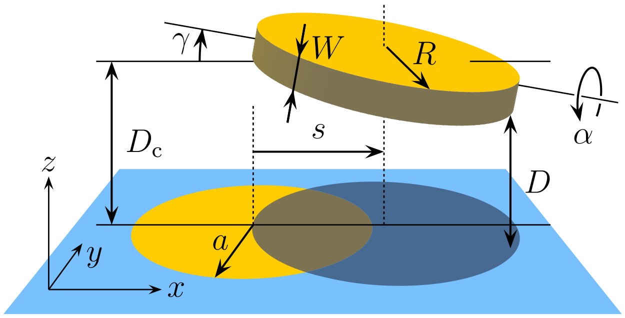

Figure S6. **Schematic of a microdisk above a circular pattern.** A microdisk of radius R and thickness W above a circular pattern of radius a on a flat substrate. D_c is the distance from the substrate to the center of the microdisk, and D is the minimal distance between the microdisk surface and the substrate (see Eq. (S2)). (D should not be confused with the symbol $\mathcal{D}$ for the diffusion coefficient.) The shift s of the center of the microdisk with respect to the center of the pattern, α, and γ describe the orientation of the microdisk with respect to the xy-plane parallel to the substrate.

and a total of seven parameters (R, W, a, D_c, s, α, and γ) fully describe the system.

It is useful to rescale all distances by the radius of the microdisk. To this end, we defined the dimensionless variables

$$\Omega = \frac{W}{R}, \quad \rho = \frac{a}{R}, \quad \Delta_c = \frac{D_c}{R}, \quad \Sigma = \frac{s}{R}. \tag{S1}$$

We also introduced the surface-to-surface distance $D = \Delta/R$ between the microdisk and the substrate. In order to relate Δ_c and Δ, we calculated the minimal value of the z coordinate of the microdisk edges for a given orientation. Geometrical considerations render

$$\Delta = \Delta_c - \frac{1}{2}\Omega\,|\cos\alpha\,\cos\gamma| - \sqrt{1-\cos^2\alpha\,\cos^2\gamma}. \tag{S2}$$

The rescaled temperature of the system is given by

$$\Gamma = R\,|(T-T_c)\,/T_c|^{\nu}\,/\xi_0^{+}, \tag{S3}$$

where T is the temperature, and T_c denotes its critical value. Here, we focus on the experimentally relevant case: the critical point is the lower critical point of a water–2,6-lutidine mixture, and only

$T < T_c$ (for which the system is homogeneous) is considered. In such circumstances, the bulk correlation length is

$$\xi(T) = \xi_{\text{non-singular}}(T) + \xi_0^+ \left[(T_c - T)/T_c\right]^{-\nu}, \tag{S4}$$

where $\xi_{\text{non-singular}}$ denotes the non-universal, non-singular part of the correlation length (which is smooth around the critical point), ξ_0^+ is a non-universal amplitude, and ν is a universal critical exponent. In the vicinity of the critical point, the diverging second term in Eq. (S4) dominates, and therefore $\Gamma \simeq R/\xi(T)$. The numerical values of the parameters T_c, ξ_0^+, and ν, characterizing the binary water–2,6-lutidine mixture, are given in Table S1.

We present the total interaction potential between the particle and the substrate as a sum of three contributions:

$$U = U_c + U_e + U_g, \tag{S5}$$

where U_c is the critical Casimir potential due to the fluctuations of a binary liquid mixture close to its critical demixing point, U_e is the screened electrostatic potential due to the surface charges and the ions in the bulk, and U_g is the gravitational potential. Estimates based on Ref. [5] indicate that critical Casimir forces are several times stronger than QED Casimir forces (dispersion forces) [6–8]. We discuss this in detail in section S1 B 3 (see Fig. S7). Consequently, we have omitted QED Casimir forces from our calculations.

1. *Gravitational potential*

The gravitational potential originates from the weight of the microdisk acting downwards together with the buoyancy force [9] acting upwards, both acting on the center of mass of the microdisk. Since these two forces are independent of position and angular configuration, the resulting potential is proportional to Δ_c and does not depend on other configurational parameters

(*viz.* α, γ, Σ). Straightforward calculations give:

$$U_{\mathrm{g}} = \pi R^4 \Omega \Delta_{\mathrm{c}} \left(\varrho_{\mathrm{p}} - \varrho_{\mathrm{l}}\right) g, \tag{S6}$$

where g is the gravity acceleration, and ϱ_{p} and ϱ_{l} are the mass densities of the microdisk and the liquid, respectively (compare Eq. (3)).

2. Critical Casimir and electrostatic potentials

In order to calculate the critical Casimir and the electrostatic contribution, we use the Derjaguin approximation. Within this approximation, the potential is given by

$$U_{\tau} = \int_A \mathfrak{u}_{\tau}\left(\ell\left(x, y\right)\right) \mathrm{d}x \, \mathrm{d}y, \tag{S7}$$

where $\tau \in \{\mathrm{c}, \mathrm{e}\}$ denotes the type of interactions (critical Casimir or electrostatic, respectively), and $\mathfrak{u}_{\tau}\left(l\right)$ is the potential per area calculated for a slab system with two homogeneous, parallel walls separated by a distance l. The function $\ell\left(x, y\right)$ is a local distance (measured in the z direction) between the substrate and the surface of the particle. The set A is an orthogonal projection of the particle onto the substrate plane.

a. Critical Casimir potential for a slab geometry In order to calculate the critical Casimir potential $\mathfrak{u}_{\mathrm{c}}$ in a slab, we consider the strong adsorption limit (*i.e.*, infinitely strong binding between the surfaces and the preferred component of the binary liquid mixture) and we ignore possible corrections to scaling. Typically, this is sufficient to capture the qualitative behaviour [10] and allows us to express the potential in terms of the universal scaling function

$$\mathfrak{u}_{\mathrm{c}}\left(l; T\right) = \frac{k_{\mathrm{B}} T_{\mathrm{c}}}{l^3} \mathcal{V}_{\mathrm{s}}\left(l \left|\left(T - T_{\mathrm{c}}\right)/T_{\mathrm{c}}\right|^{\nu} / \xi_0^{+}\right), \tag{S8}$$

where k_{B} is the Boltzmann constant, and $\mathrm{s} \in \{\mathrm{same}, \mathrm{opposite}\}$ denotes whether the boundary conditions on the two surfaces are the same or opposite (*i.e.*, whether the two surfaces prefer the same or different components of the binary liquid mixture). For $T \lesssim T_{\mathrm{c}}$, the argument of

**quantity**	**symbol**	**value**
acceleration of Earth gravity	g	$9.8\,\mathrm{m/s^2}$
mass density of the particles	ϱ_p	$2203\,\mathrm{kg/m^3}$
mass density of the critical water–2,6-lutidine mixture	ϱ_l	$976.7\,\mathrm{kg/m^3}$
critical temperature of the water–2,6-lutidine mixture	T_c	$307\,\mathrm{K}^*$
critical exponent ν within the 3D Ising universality class	ν	0.63^*
vacuum permittivity	ε_0	$8.854 \times 10^{-12}\,\mathrm{F/m}$
relative permittivity of critical water–2,6-lutidine mixture	ε	7^*
Boltzman constant	k_B	$1.381 \times 10^{-23}\,\mathrm{J/K}$
correlation length of critical water–2,6-lutidine mixture	ξ_0^+	$0.2\,\mathrm{nm}^*$
scaled surface charge density	$\sigma/\sqrt{\varepsilon}$	$0.0015\,\mathrm{C/m^2}$
Debye length	λ_D	$10\,\mathrm{nm}$
radius of the microdisk	R	$1.2\,\mathrm{\mu m}$
thickness of the microdisk	W	$0.4\,\mathrm{\mu m}$

Table S1. **Symbols and their numerical values used in the calculations.** The upper part of the table lists universal and material constants and the bottom part contains values relevant for the experiment. Other parameters (such as the reduced temperature Γ and the size a of the pattern on the substrate) and the configuration of the microdisk (*viz.* Δ_c, Σ, α, and γ) have been varied in the course of the calculations. Since we used the reduced temperature Γ rather than T (as done for describing the experiments), there was no need to use T_c, ξ_0^+, and ν in our calculations (although these values were necessary to estimate the value of Γ). Also, because we used the rescaled surface charge density $\sigma/\sqrt{\varepsilon}$, the numerical value of the relative permittivity ε was not needed in the caluclations. Nevertheless, for the sake of completeness, we present their values in the table but mark them with an asterisk (*).

the scaling function $\mathcal{V}_\mathrm{s}$ in Eq. (S8) is $l\left|(T-T_\mathrm{c})/T_\mathrm{c}\right|^\nu/\xi_0^+ \simeq l/\xi(T)$ (see Eq. (S4)). The universal scaling functions $\mathcal{V}_\mathrm{same}$ and $\mathcal{V}_\mathrm{opposite}$ for the experimentally relevant 3D Ising universality class have been estimated via Monte Carlo simulations [11]; in our calculations we used the formulae from Ref. [12] fitted to the numerical results.

b. Electrostatic interaction potential for a slab geometry For the electrostatic potential, we have assumed a constant surface charge σ, the same on the substrate and on the microdisk. This leads to

$$\mathrm{u_e}\left(l\right) = \frac{\sigma^2}{4\pi\varepsilon_0\varepsilon}\mathrm{e}^{-l/\lambda_\mathrm{D}}, \tag{S9}$$

where λ_D denotes the Debye length, ε_0 is the absolute permittivity of vacuum, and ε denotes the relative permittivity of the water–2,6-lutidine mixture. Thus, the energy of the electrostatic interaction depends on two variables: the rescaled surface charge density $\sigma/\sqrt{\varepsilon}$ and the Debye length λ_D. Since an experimental determination of these parameters was not possible, we have tuned them to obtain a qualitative agreement between theoretical calculations and experiments for (i) the probability $\mathcal{P}_{||}$ of the parallel configuration above a circular pattern (Fig. 2(b)) and (ii) for the trajectories above the triangular pattern (Fig. 5(b,f)). The estimated values of $\sigma/\sqrt{\varepsilon}$ and λ_D are listed in Table S1. (We note that a similar level of agreement between the numerical calculations and the experimental data can be achieved for a wide range of σ and λ_D. Moreover, the results of the calculation are influenced by inaccuracies due to the Derjaguin approximation. Therefore, our estimates for the surface charge density and the Debye length cannot be considered as faithful approximations of their true values.)

3. QED Casimir interactions

We calculate the QED Casimir interaction in both cases, i.e., for a microdisk suspended on (i) a gold-coated silica substrate and (ii) for an uncoated silica substrate, in an infinite-plane geometry, i.e., we assume that the microdisk exhibits an infinite lateral extension and that the flake is always parallel to the substrate. For the calculation of the interaction, we follow the same method as used in Ref. 13, which is based on the corresponding method described in Parsegian's book [14]. The spatial arrangement of the materials constituting the system is represented in Fig. S7.

The QED Casimir interaction, in a planar geometry, depends on the separation distance h, which represents the thickness of the water-2,6 luditine layer separating the microdisk from the substrate, as

$$F_{\mathrm{QED}}(h) = -\frac{\mathrm{d}U_{\mathrm{QED}}}{\mathrm{d}h} S, \tag{S10}$$

where U_{QED} is the QED Casimir free energy per area of the system with $S = \pi R^2$ as the area of the microdisk (Ref. 14). The force depends on the thicknesses of the layers (see Figure S7) and on the dielectric properties of the materials involved. Explicitly, the QED Casimir interaction can be expressed in terms of Hamaker's functions as [14]

$$U_{\mathrm{QED}}(h) \;=\; -\frac{A_{2/3}(h)}{12\pi^2 h^2} - \frac{A_{1/3}(h+a_1)}{12\pi(h+a_1)^2} - \frac{A_{2/4}(h+b)}{12\pi(h+b)^2} - \frac{A_{1/4}(h+a_1+b)}{12\pi(h+a_1+b)^2}, \tag{S11}$$

where $A_{2/3}$, $A_{1/3}$, $A_{2/4}$, and $A_{1/4}$ are Hamaker's functions, a_1 is the thickness of the gold layer deposited on the substrate, and b is the thickness of the microdisk. In the simpler case of a bottom slide of uncoated silica, instead, the QED Casimir interaction can be written as

$$U_{\mathrm{QED}}(h) \;=\; -\frac{A_{5/3}(h)}{12\pi h^2} - \frac{A_{5/4}(h+b)}{12\pi(h+b)^2}, \tag{S12}$$

where $A_{5/3}$ and $A_{5/4}$ correspond to Hamaker's functions.

Following Ref. 14, the Hamaker functions for the free energy of the layered systems in Fig. S7 (with the relevant interfaces numbered accordingly, where WL stands for the water–2,6-lutidine

mixture, SiO_2 for silica, and Au for gold layers) are:

$$\begin{aligned}
A_{2/3}(h) &\approx \frac{3}{2}k_\mathrm{B}T\sum_{n=0}^{\infty}{}'\Delta_{\mathrm{Au-WL}}\Delta_{\mathrm{SiO_2-WL}}R_n(h),\\
A_{1/3}(h+a_1) &\approx \frac{3}{2}k_\mathrm{B}T\sum_{n=0}^{\infty}{}'\Delta_{\mathrm{SiO_2-Au}}\Delta_{\mathrm{SiO_2-WL}}R_n(h+a_1),\\
A_{2/4}(h+b) &\approx \frac{3}{2}k_\mathrm{B}T\sum_{n=0}^{\infty}{}'\Delta_{\mathrm{Au-WL}}\Delta_{\mathrm{WL-SiO_2}}R_n(h+b),\\
A_{1/4}(h+a_1+b) &\approx \frac{3}{2}k_\mathrm{B}T\sum_{n=0}^{\infty}{}'\Delta_{\mathrm{SiO_2-Au}}\Delta_{\mathrm{WL-SiO_2}}R_n(h+a_1+b),\\
A_{5/3}(h) &\approx \frac{3}{2}k_\mathrm{B}T\sum_{n=0}^{\infty}{}'\Delta_{\mathrm{SiO_2-WL}}\Delta_{\mathrm{SiO_2-WL}}R_n(h),\\
A_{5/4}(h+b) &\approx \frac{3}{2}k_\mathrm{B}T\sum_{n=0}^{\infty}{}'\Delta_{\mathrm{SiO_2-WL}}\Delta_{\mathrm{WL-SiO_2}}R_n(h+b).
\end{aligned} \tag{S13}$$

Concerning these equations we note that $\Delta_{\mathrm{a-b}}$ with $\mathrm{a,b} \in \{\mathrm{SiO_2, Au, WL}\}$ are defined as

$$\Delta_{\mathrm{a-b}} = \frac{\epsilon_\mathrm{a} - \epsilon_\mathrm{b}}{\epsilon_\mathrm{a} + \epsilon_\mathrm{b}} = -\Delta_{\mathrm{b-a}}, \tag{S14}$$

where ϵ_a and ϵ_b are the dielectric functions of the materials forming the interfaces, while R_n accounts for the relativistic retardation. The prime ($\prime$) in the sum indicates that the contribution for $n = 0$ has to be multiplied [14, 15] by a factor 1/2. The dielectric functions entering into the definition of $\Delta_{\mathrm{a-b}}$ have to be calculated at the complex frequencies $i\omega_\mathrm{n}$ specified further below, for each of the materials involved (gold, silica, water–2,6-lutidine) in the layered system. R_n is the relativistic screening function defined as $R_n = (1 + r_n)\, e^{-r_n(z)}$, where z indicates the generic argument of R_n appearing in Eq. S13, with $r_n(z) = 2\tau_n(z)\,\omega_n$ for a distance z between the involved interfaces. This function quantifies the relevance of the retardation effects. The quantity $\tau_n(z)$ is the minimum time an electric field, generated by the fluctuating dipole, takes to travel the distance z and therefore is given by $\tau_n(z) = \frac{z}{c\sqrt{\epsilon_{WL}(i\omega_n)}}$ where c is the speed of light in vacuum, while ω_n are the frequencies $\omega_n = \frac{2\pi k_\mathrm{B}T}{\hbar}n \simeq n \times 2.4678 \times 10^{14}\,\mathrm{rad\,s^{-1}}$ for the temperatures involved in our experiment. If the minimum retardation time $2\tau_n(z)$ is small with respect to the inverse

of the electromagnetic field angular frequency ω_n, one has $r_n(z) \ll 1$ and the retardation effects are negligible. In general, $r_n(z) \simeq z \times n \times 1.19 \times 10^6$ taking $\sqrt{\epsilon_{WL}(i\omega_n)} \approx n_{WL} \approx 1.38$, as in Ref. 15. The parametrization of the dielectric functions used to compute the Hamaker's functions (Eqs. (S13)) are the following: for water–2,6-lutidine and silica we follow Ref. 15, while for gold we follow Ref. 14. This choice for the dielectric functions has also been used in Ref. 13.

4. Numerical implementation

In order to calculate the critical Casimir and the electrostatic potential, we wrote a computer program in C++ to numerically compute the corresponding integrals (see Eq. (S7)). The integration was separately done for the rim and the side of the microdisk (note that only the side facing the substrate is relevant within the Derjaguin approximation). Each two–dimensional integral was calculated numerically as two nested one–dimensional integrals using the GSL Library [16]. In order to enhance the accuracy, we calculated separately the integrals over the regions with the same and the opposite boundary conditions (see Ref. [12] for details).

5. Example

In Fig. S8, we show two examples of the force and of the torque acting on the disk oriented parallel to the substrate (*i.e.*, $\alpha = 0$ and $\gamma = 0$) for the same parameters as in Fig. 1 of the main text. We computed the force by numerically differentiating the interaction potential with respect to the disk height D. Similarly, the torque τ_γ was determined numerically by differentiating with respect to the angle γ. This figure shows that both the force and the torque become an order of magnitude stronger as the temperature difference $\Delta T = T - T_c$ approaches $\Delta T = 0$, *i.e.*, the critical point. Since the torque is positive for $\gamma > 0$ and negative for $\gamma < 0$, the parallel configuration at the equilibrium height is stable with respect to rotations.

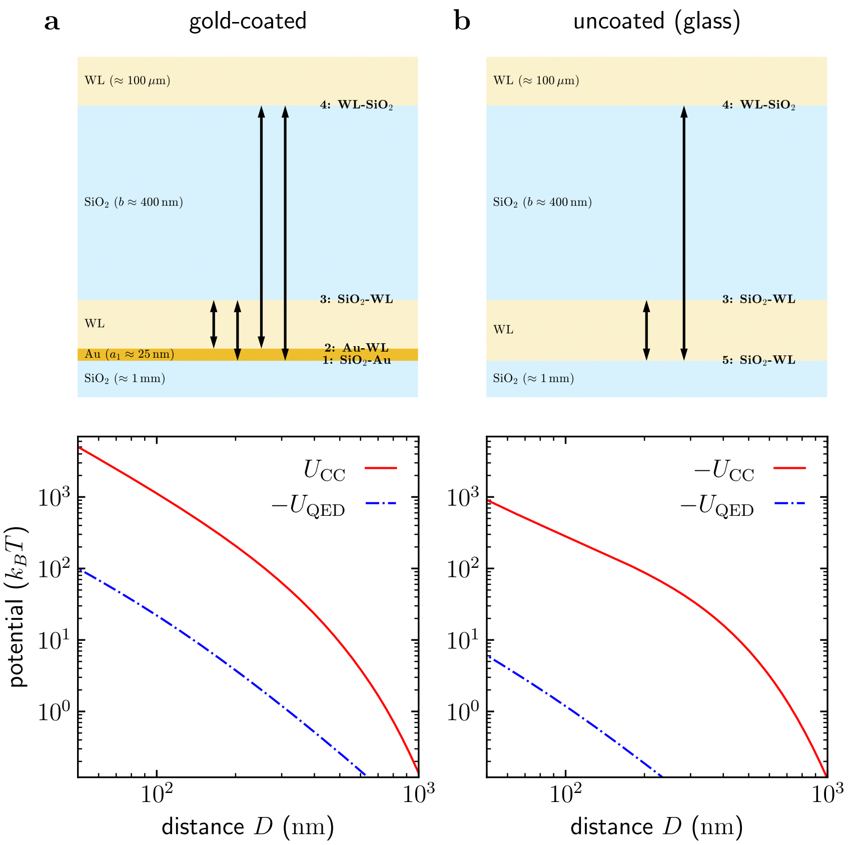

Figure S7. **Critical Casimir vs QED Casimir potentials.** Critical Casimir and QED Casimir potentials between a disk and a substrate expressed in units of k_BT as functions of the distance from the substrate for (**a**) gold-coated and (**b**) uncoated (glass) substrates. The cartoons in the upper row show the parameters used for calculating the QED Casimir potential. The system has a planar, layered geometry: the bottom silica layer and the top water-lutidine layer are taken to be semi-infinitely large. The bottom, semi-infinite silica layer is either gold-coated (**a**) or uncoated (**b**). The top semi-infinite water-lutidine film is next to a silica layer representing the microdisk. The intermediate water-lutidine layer acts as a separating medium. There are four distinct interfaces formed by the media for the gold-coated substrate, and three for the uncoated substrate. In each panel, the labels naming each interface are written to the right of the black arrows, for each corresponding interface. The black arrows connecting the interfaces on different sides of the separating medium represent specific Hamaker's functions (see Eq. (S13)). The lower panels tell that the critical Casimir potential is stronger than the QED Casimir potential by a factor of ca. 100. Source data are provided as a Source Data file.

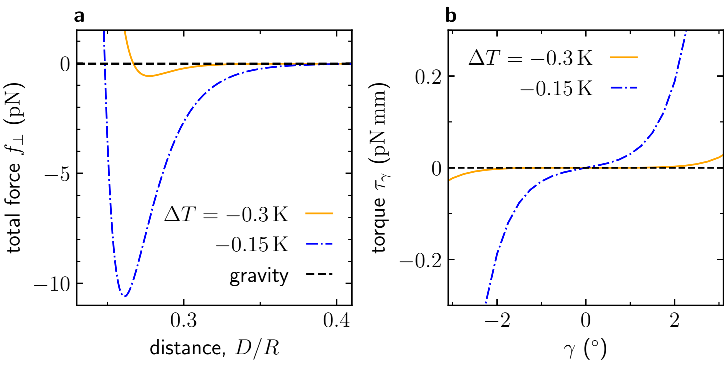

Figure S8. **Examples of forces and torques.** (**a**) Force $f_\perp$ acting perpendicularly to the substrate as a function of the disk height for a disk oriented parallel to the substrate and for two temperature differences $\Delta T = T - T_c$. The gravitational force (ca. $-0.022\,\mathrm{pN}$) is shown with the horizontal dashed line. (**b**) Torque τ_γ for rotation around the angle γ as a function of γ (see Fig. S6) for two values of ΔT and for the disk height D_{min} at which $f_\perp = 0$. The dashed line corresponds to zero torque. The patch size is $a/R = 1$ in both plots. Source data are provided as a Source Data file.

C. Analysis of microdisk configurations

In order to study the configurations of the microdisk, we calculated the minimum of the potential U with respect to Δ_c and Σ for a fixed value of γ and for $\alpha = 0°$. We denote the value of the potential at the minimum by U_{min}, and the corresponding values of the rescaled surface-to-surface distance and of the lateral shift, for which the minimum is attained, by Δ_{min} and Σ_{min}, respectively. It turns out that the constraint $\alpha = 0°$ is not limiting our analysis. At $\alpha = 0°$, the potential always exhibits a local minimum with respect to α, and the possible minima for $\alpha \neq 0°$ appear for α almost equal to 90°. They are close to, but not as deep as, the minimum occurring for the perpendicular configuration, *i.e.*, $\alpha = 0°$ (see below).

Typical plots of U_{min}, Δ_{min}, and Σ_{min} are presented in Fig. S9 (we chose $a/R = \rho = 0.65$ and $R/\xi \simeq \Gamma = 10$ in this plot). For $\gamma = 0°$, *i.e.*, if the microdisk is parallel to the substrate, U_{min} and Δ_{min} correspond to a global maximum as functions of γ. For $\gamma = 0°$, the value of Σ_{min} cannot

be determined uniquely because it is a multivalued function of γ. This is the case because the potential does not depend on Σ as long as $|\Sigma| < |1-\rho|$, *i.e.*, if the orthogonal projection of the microdisk onto the substrate either covers the pattern completely (for $\rho < 1$) or if it is completely inside the pattern (for $\rho > 1$). In Fig. S9(c), all these possible values of Σ_{min} correspond to the vertical segment of the plot at $\gamma = 0°$. We note, however, that this behavior is an artifact of the Derjaguin approximation. In the vicinity of $\gamma = 0°$, we expect that for U_{min} the actual lateral shift Σ_{min} of the microdisk varies continuously, albeit rapidly, as a function of γ.

Upon increasing γ from 0° (*i.e.*, tilting the microdisk clockwise), the surface-to-surface distance Δ_{min} decreases and the center of the microdisk moves to the left ($\Sigma_{\text{min}} < 0$). At the same time, the potential U_{min} is decreasing and has a minimum for $\gamma = \gamma_{||}$. At this point, the potential U exhibits a minimum with respect to all configurational parameters. Since, typically, $\gamma_{||}$ is close to 0°, we identify this minimum with the parallel configuration observed in the experiment.

Upon further increasing γ, we observe first a shallow minimum of the lateral shift Σ_{min} (at this point the microdisk has the largest lateral shift from the center of the pattern on the substrate), followed by a very shallow minimum of the surface-to-surface distance Δ_{min} (at this point the distance from the microdisk to the substrate is the smallest). At the same time, U_{min} increases, exhibits a local maximum, and then decreases. For $\gamma = \gamma_{\perp}$, there is a second minimum with respect to all configurational parameters of U. The value of $\gamma_{\perp}$ is only slightly below 90° and $\Sigma_{\text{min}}(\gamma_{\perp})$ is only slightly below zero. We identify this minimum with the perpendicular configuration of the microdisk observed in the experiment.

For $\gamma = 90°$ (and $\rho < 1$), the microdisk is located exactly at the center of the pattern on the substrate. This is a local maximum (with respect to γ) of U_{min} and Δ_{min} (barely visible in Fig. S9). Around this point, U_{min} as a function of γ has a shape similar to the letter "ω" with two minima for $\gamma = \gamma_{\perp}$ and $\gamma = 180° - \gamma_{\perp}$ with $\gamma_{\perp} \lesssim 90°$, and a cusp in the maximum for $\gamma = 90°$.

Due to the symmetry of mirror reflection of the system, the potentials for $\gamma < 0°$ and for

$\gamma > 90°$ are mirror reflections of those for $\gamma > 0°$ and $\gamma < 90°$, respectively. While $U_{\rm min}$ and $\Delta_{\rm min}$ as functions of γ are symmetric around these two points, the lateral shift $\Sigma_{\rm min}$ is antisymmetric.

We have checked numerically that the potential U has the two local minima (described above) with respect to all configurational parameters for a wide range of values for Γ and ρ. The depth of these minima of the potential strongly depends on the values of the parameters — typically, for large ρ, the potential is weaker for the minimum corresponding to the parallel configuration, and for small ρ, it is weaker for the perpendicular configuration. For certain ranges of parameters the values of the potential for these minima can differ by several orders of magnitude. Therefore, we expect a first-order morphological transition between the parallel and the perpendicular configurations, in line with the experimental observations. We discuss the methods used to study this transition in the following section.

We note that the potential U exhibits two additional local minima, for which the microdisk is located far away from the pattern on the substrate. In these configurations, the electrostatic and the critical Casimir repulsion are balanced by the gravitational attraction. Apart from the limiting case $\rho \to 0$, these minima are much more shallow than in the configurations discussed above. A detailed analysis of these minima and the limit of a small size of the pattern is beyond the scope of the present study.

D. Monte Carlo simulations for microdisk orientation

In order to determine the probabilities of encountering the colloidal particle in its parallel or perpendicular configuration (see Fig. 2c of the main text) one can, in principle, integrate the Boltzman factor $\exp\left[-U/\left(k_{\rm B}T\right)\right]$ over those parts of the parameter space which are assigned to these two configurations. However, in our case this approach is not feasible. On the one hand, the energy landscape around each minimum is too shallow to approximate the integrals by taking into

account only the direct vicinity of both minima. On the other hand, the integration over large parts of the parameter space can only be carried out numerically and requires to calculate the potential at many points, thus being time consuming.

In order to avoid the above problems, we used Monte Carlo (MC) simulations to estimate the probabilities for the configurations of the colloidal particle. We assigned a statistical weight to each configuration of the microdisk:

$$w\left(\Delta, \Sigma, \alpha, \gamma\right) = \left|\Sigma \cos\alpha\right| \exp\left[-U/\left(k_{\mathrm{B}}T\right)\right], \tag{S15}$$

which takes into account, beyond the Boltzmann factor, the volume of the parameter space; we present derivation of this factor section S1 D 1. We note that in the simulation we used the reduced surface-to-surface distance Δ as a parameter rather than the reduced height Δ_{c} of the center of the microdisk above the substrate. The parameter Δ is more versatile since, as we have checked, it usually has similar values for both potential minima (*i.e.*, for the parallel and the perpendicular configurations).

For given values of the reduced temperature Γ and of the size $\rho = a/R$ of the pattern, we proceeded with simulations and analysis as describe in sections S1 D 2 to S1 D 4.

1. *Derivation of the statistical weight of configurations*

In order to preserve detailed balance in MC simulations, the weight assigned to each configuration must be proportional to the probability of finding a particle in this configuration. We obtain this probability within the canonical ensemble, which requires to determine the Hamiltonian of a particle.

Since the disk particle is a rigid body, it has six degrees of freedom. Accordingly, we need six variables to fully describe its configuration. We use the following procedure: Initially the disc is located such that its center is in the origin of the coordinate system with its circular surfaces

parallel to the xy plane. Then, we rotate this disk around the z axis by an angle η, around the axis x by an angle α, and around the axis y by an angle γ. Next, the disk is translated by a vector $\boldsymbol{w} = (s, D_\mathrm{c}, 0)^\mathrm{T}$, and finally rotated around the z axis by an angle ω. This way the configuration of the disk is described with six parameters $\boldsymbol{X} = (\eta, \alpha, \gamma, s, D_\mathrm{c}, \omega)^\mathrm{T}$. Four of them have already been introduced and are denoted in Fig. S6. The additional two angles η and ω are necessary only to define the kinetic energy of the system; the potential does not depend on them due to symmetry reasons.

We start the derivation of the probability by calculating the kinetic energy of the particle. To this end we parameterise the disk in its initial state by using cylindrical coordinates r, ϕ, and z, *i.e.*,

$$\boldsymbol{r}_0 = (r\cos\phi, r\sin\phi, z)^\mathrm{T}\,, \tag{S16}$$

with $0 \leqslant r \leqslant R$, $0 \leqslant \phi < 2\pi$, and $-W/2 \leqslant z \leqslant W/2$. After the above described transformations, the parametrisation of the disk is given by

$$\boldsymbol{r} = \mathrm{R}_z(\omega)\left[\mathrm{R}_y(\gamma)\,\mathrm{R}_x(\alpha)\,\mathrm{R}_z(\eta)\,\boldsymbol{r}_0 + \boldsymbol{w}\right], \tag{S17}$$

where R_x, R_y, and R_z denote the matrices of rotation around the x, y, and z axis, respectively. The kinetic energy of the particle is given by

$$T = \int_0^R r\mathrm{d}r \int_0^{2\pi} \mathrm{d}\phi \int_{-W/2}^{W/2} \mathrm{d}z\, \frac{1}{2}\varrho_\mathrm{p}\, \dot{\boldsymbol{r}}^\mathrm{T}\dot{\boldsymbol{r}}, \tag{S18}$$

where $\varrho_\mathrm{p} = M/(\pi R^2 W)$ is the mass density of the disk, M denotes its mass, and the dot denotes the time derivative. After straightforward but rather tedious calculations we obtain the kinetic

energy

$$T = \frac{MR^2}{4}\dot{\eta}^2 + \frac{M}{24}\left(3R^2 + W^2\right)\dot{\alpha}^2 + \frac{M}{48}\left[9R^2 + W^2 + \left(W^2 - 3R^2\right)\cos 2\alpha\right]\dot{\gamma}^2 + \frac{M}{2}\dot{s}^2$$
$$+ \frac{M}{2}\dot{D}_{\mathrm{c}}^{\ 2} + \frac{M}{96}\left[15R^2 + 3W^2 + \left(3R^2 - W^2\right)\left(4\cos^2\alpha\cos^2\gamma - 1\right) + 48s^2\right]\dot{\omega}^2$$
$$- \frac{MR^2}{2}\sin\alpha\,\dot{\eta}\dot{\gamma} + \frac{MR^2}{2}\cos\alpha\cos\gamma\,\dot{\eta}\dot{\omega} - \frac{M}{12}\left(3R^2 + W^2\right)\sin\gamma\,\dot{\alpha}\dot{\omega}$$
$$+ \frac{M}{24}\left(W^2 - 3R^2\right)\cos\gamma\sin 2\alpha\,\dot{\gamma}\dot{\omega} = \frac{1}{2}\dot{\boldsymbol{X}}^{\mathrm{T}}\mathbb{A}\dot{\boldsymbol{X}}, \quad \text{(S19)}$$

where $\dot{\boldsymbol{X}} = \left(\dot{\eta}, \dot{\alpha}, \dot{\gamma}, \dot{s}, \dot{D}_{\mathrm{c}}, \dot{\omega}\right)^{\mathrm{T}}$ and $\mathbb{A}$ is the 6×6 matrix as defined via Eq. (S19).

The second step of the derivation is to determine the Hamiltonian of the disk particle. To this end we introduce generalised momenta

$$\boldsymbol{\Pi} = \left(\Pi_\eta, \Pi_\alpha, \Pi_\gamma, \Pi_s, \Pi_D, \Pi_\omega\right)^{\mathrm{T}} = \left(\frac{\partial T}{\partial\dot{\eta}}, \frac{\partial T}{\partial\dot{\alpha}}, \frac{\partial T}{\partial\dot{\gamma}}, \frac{\partial T}{\partial\dot{s}}, \frac{\partial T}{\partial\dot{D}_{\mathrm{c}}}, \frac{\partial T}{\partial\dot{\omega}}\right)^{\mathrm{T}}, \quad \text{(S20)}$$

so that by using Eq. (S19) the resulting Hamiltonian is

$$\mathcal{H} = \frac{1}{2}\boldsymbol{\Pi}^{\mathrm{T}}\mathbb{A}^{-1}\boldsymbol{\Pi} + U\left(D_{\mathrm{c}}, s, \alpha, \gamma\right). \quad \text{(S21)}$$

In the third step we calculate the probability for four relevant variables. In the canonical ensemble the probability p for all 12 variables (6 configuration variables and 6 momenta) is proportional to $\exp\left(-\beta\mathcal{H}\right)$. Therefore one has

$$p\left(D_{\mathrm{c}}, s, \alpha, \gamma\right)$$
$$\sim \int_{-\infty}^{\infty}\mathrm{d}\Pi_\alpha\int_{-\infty}^{\infty}\mathrm{d}\Pi_\gamma\int_{-\infty}^{\infty}\mathrm{d}\Pi_\eta\int_{-\infty}^{\infty}\mathrm{d}\Pi_s\int_{-\infty}^{\infty}\mathrm{d}\Pi_D\int_{-\infty}^{\infty}\mathrm{d}\Pi_\omega\int_0^{2\pi}\mathrm{d}\eta\int_0^{2\pi}\mathrm{d}\omega\exp\left(-\beta\mathcal{H}\right)$$
$$= \left(2\pi\right)^5\sqrt{\det\mathbb{A}}\,\exp\left(-\beta U\right). \quad \text{(S22)}$$

Since $\det\mathbb{A} = M^6R^2\left(3R^2 + W^2\right)^2 s^2\cos^2\alpha/288$, we have

$$p\left(D_{\mathrm{c}}, s, \alpha, \gamma\right) = \mathcal{A}\left|s\cos\alpha\right|\exp\left(-\beta U\right), \quad \text{(S23)}$$

where $\mathcal{A}$ is a normalisation constant, independent of the variables D_{c}, s, α, and γ.

Finally, we change the variables of the probability from $(D_{\mathrm{c}}, s, \alpha, \gamma)$ to $(\Delta, \Sigma, \alpha, \gamma)$ (see Eqs. (S1) and (S2)). A straightforward calculation using Eq. (S2) shows that the Jacobian of this transformation equals R^2. Hence, the weight in Eq. (S15) is proportional to the probability (S23), as required for detailed balance.

2. Preparation of simulations

We start by identifying two local minima of the potential U. We denote the values of the parameters for which the potential is minimal by $\Delta_{\parallel}$, $\Sigma_{\parallel}$, $\alpha_{\parallel}$, and $\gamma_{\parallel}$ ($\Delta_{\perp}$, $\Sigma_{\perp}$, $\alpha_{\perp}$, and $\gamma_{\perp}$) for the parallel (perpendicular) configuration. After that, we estimate the value of the energy barrier U_{b} of the potential between these minima. Since a precise calculation of this quantity is quite time consuming, we use the following approximation:

$$U_{\mathrm{b}} = \max_{0<t<1} U\left((1-t)\Delta_{\parallel} + t\Delta_{\perp}, (1-t)\Sigma_{\parallel} + t\Sigma_{\perp}, (1-t)\alpha_{\parallel} + t\alpha_{\perp}, (1-t)\gamma_{\parallel} + t\gamma_{\perp}\right), \qquad \text{(S24)}$$

which is the maximum of the potential along the linear interpolation in the parameter space between the parallel and the perpendicular configurations. We calculate this maximum numerically. We note that this procedure provides an upper bound for the energy barrier.

Next, we calculate $\Delta_{\max}$ and $\Delta_{\min}$, which are the limiting values for the reduced surface-to-surface distance. (For the Monte Carlo moves discussed below, we choose Δ according to a uniform probability for the region between $\Delta_{\min}$ and $\Delta_{\max}$; see Eq. (S26).) We take them initially to be equal to the largest and the smallest values out of $\left\{\Delta_{\parallel}, \Delta_{\perp}\right\}$, respectively. Then, we keep increasing $\Delta_{\max}$ (by multiplying it by 1.1) and decreasing $\Delta_{\min}$ (by multiplying it by 0.9) until the following inequalities are satisfied (see Eq. (S15)):

$$w\left(\Delta_{\max}, \Sigma_{\parallel}, \alpha_{\parallel}, \gamma_{\parallel}\right) < 0.001 w\left(\Delta_{\parallel}, \Sigma_{\parallel}, \alpha_{\parallel}, \gamma_{\parallel}\right) > w\left(\Delta_{\min}, \Sigma_{\parallel}, \alpha_{\parallel}, \gamma_{\parallel}\right), \qquad \text{(S25a)}$$

$$w\left(\Delta_{\max}, \Sigma_{\perp}, \alpha_{\perp}, \gamma_{\perp}\right) < 0.001 w\left(\Delta_{\perp}, \Sigma_{\perp}, \alpha_{\perp}, \gamma_{\perp}\right) > w\left(\Delta_{\min}, \Sigma_{\perp}, \alpha_{\perp}, \gamma_{\perp}\right), \qquad \text{(S25b)}$$

where $w(\Delta, \Sigma, \alpha, \gamma)$ is given by Eq. (S15). The choice of these conditions is motivated by the assumption that the configurations, with their statistical weight being smaller by a factor 0.001 than the weight of the minimal configurations, have such a low probability of occurrence that ignoring them does not affect the calculated probabilities appreciably.

We finish the initialization of the simulation by choosing the initial configuration of the colloidal particle randomly with uniform probability out of all configurations fulfilling the conditions

$$\Delta_{\min} < \Delta < \Delta_{\max}, \quad -\rho < \Sigma < \rho, \quad 0^\circ \leqslant \alpha, \gamma < 360^\circ, \quad U < U_{\mathrm{b}} + 2\, k_{\mathrm{B}} T. \tag{S26}$$

We note that we allow for $\Sigma = s/R < 0$; this makes it easier to properly probe the configurations around $\Sigma = 0$ in our simulations.

3. Running simulations

Each step of the simulation consists of two phases. First, we attempt a Monte Carlo move by modifying the configuration slightly. We modify Δ, Σ, α, and γ by a random real number between -0.01 and 0.01 (with a uniform probability density). If, after the move, the conditions in Eq. (S26) are not satisfied, it is rejected. Otherwise, we calculate the weight w_{new} of the new configuration by using Eq. (S15), and compare it with the weight w of the unchanged configuration. Following the Metropolis algorithm [17], we accept the move with the probability

$$p = \min \{ w_{\mathrm{new}}/w, 1 \} . \tag{S27}$$

Within each step of the Monte Carlo simulation, we repeat the attempts of the small modifications 32 times, and then we attempt one large change of the configuration — we choose a completely new configuration (with uniform probability) satisfying the conditions in Eq. (S26) and accept or reject this move by following the same rule as in Eq. (S27). Small changes are necessary for the particle to probe the parameter space around the local minimum, whereas large changes make sure that the simulation can access both minima.

In all Monte Carlo simulation, we have undertaken 2000 steps as described above. We have checked that this number of steps was sufficient for the system to reach thermal equilibrium.

4. Analysis of simulation results

In order to determine the configuration state of the microdisk, we first calculate the value of the potential U and, if it exceeds U_{b}, we consider the particle to be unbound from the substrate and thus we do not count this case. Otherwise, we look at the angle $0° \leqslant \psi(\alpha, \gamma) < 90°$ between the axis of rotational symmetry of the microdisk and the direction normal to the substrate. We compare ψ with the angles $\gamma_{\|}$ and $\gamma_{\perp}$ characterizing the parallel and perpendicular configurations, respectively; since $\alpha_{\|} = \alpha_{\perp} = 0°$, we have $\psi\left(\alpha_{\|}, \gamma_{\|}\right) = \gamma_{\|}$ and $\psi\left(\alpha_{\perp}, \gamma_{\perp}\right) = \gamma_{\perp}$. Whichever of these two angles, $\gamma_{\|}$ or $\gamma_{\perp}$, is closer to ψ determines the state of the microdisk configuration. In practice, since $\cos\psi = |\cos\gamma\cos\alpha|$, the microdisk is in the parallel configuration if

$$|\cos\gamma\cos\alpha| > \cos\left(\frac{\gamma_{\|} + \gamma_{\perp}}{2}\right), \tag{S28}$$

and in the perpendicular configuration otherwise.

In order to estimate the probability of the microdisk being in the parallel or perpendicular configuration for a given reduced temperature $\Gamma \simeq R/\xi$ and size $\rho = a/R$ of the pattern, we carried out the Monte Carlo simulation described above independently for 200 particles. We calculated the probability shown in Fig. 2c by recording the number of parallel configurations, taking the final configuration from each independent run.

5. Example

In Fig. S10 we present typical results of the Monte Carlo simulations. Each particle is represented by a segment with its length equal to the microdisk diameter (which is 2 in units of the microdisk radius) with its center located at the microdisk center. The tilt angle $\phi(\alpha, \gamma)$ of

each segment illustrates the microdisk angular configuration. We determined ϕ by rotating the microdisk by the smallest possible angle around an axis normal to the substrate and intersecting the center of the microdisk until the angle α, describing a new, rotated configuration, is zero. We take ϕ to be equal to the angle γ in this configuration. It can be shown that $\phi = \pm\psi$, where ψ is the angle which we used to determine whether the microdisk is in the parallel or the perpendicular configuration (see section S1 C), where

$$\sin\phi\,(\alpha,\gamma) = \begin{cases} -\sqrt{1-\cos^2\alpha\cos^2\gamma} & \text{for } \sin 2\gamma \geqslant 0, \\ \sqrt{1-\cos^2\alpha\cos^2\gamma} & \text{for } \sin 2\gamma < 0, \end{cases} \qquad -90^\circ \leqslant \phi \leqslant 90^\circ. \tag{S29}$$

We emphasize that this microdisk rotation is done solely for illustrative purposes to make the configurations of the microdisks better visible for the graphs presented in Fig. S10.

As shown in Fig. S10, if the size a of the pattern on the substrate is small, all microdisks are in the parallel configuration (see Fig. S10(a)). Upon increasing a, a few microdisks acquire the parallel configuration (see the segment on the right side in Fig. S10(b)). A further increase of a leads to an increasing number of microdisks in the parallel configuration (see Fig. S10(c)) until only a few microdisks remain in the perpendicular configuration for sufficiently large a (see Fig. S10(d)).

E. Critical Casimir ratchet

In this section, we describe the methods we have used to calculate the potential free energy landscape of a microdisk above a surface with a triangular pattern (Fig. 5c) and the corresponding averaged trajectories (Fig. 5d).

1. Free energy landscape

Based on our experimental observations, we assume that the microdisk above a surface with triangular patterns is always in a parallel configuration. Although this configuration involves small

disk tilting, for simplicity, we assume the microdisk to be exactly parallel to the substrate (*i.e.*, $\alpha = 0$ and $\gamma = 0$) (see section S1 C). Given that the microdisk in the parallel configuration is tilted only slightly, this assumption should not influence the interaction potential significantly. Within the Derjaguin approximation, the interaction energy therefore is given by

$$U_{\parallel}(x,y,z) = \pi R^2 W(\varrho_{\mathrm{p}} - \varrho_{\mathrm{l}})\, gz + \pi R^2\, \mathfrak{u}_{\mathrm{e}}(z - W/2) + \frac{k_{\mathrm{B}} T_{\mathrm{c}}}{(z - W/2)^3}\left[A_{\mathrm{same}}\mathcal{V}_{\mathrm{same}}\big((z - W/2)/\xi\big) + A_{\mathrm{opposite}}\mathcal{V}_{\mathrm{opposite}}\big((z - W/2)/\xi\big)\right], \quad \text{(S30)}$$

where x, y, and z denote the position of the center of the microdisk. In Eq. (S30), the first term describes the gravitational energy (see Eq. (S6)), the second term is due to the electrostatic energy (see Eq. (S9)), and the last term describes the critical Casimir energy (see Eq. (S8) and below). The distance $z - W/2$ is the surface-to-surface distance between the microdisk and the substrate. A_{same} (A_{opposite}) is the area of the projection of the microdisk onto the substrate which prefers the same (opposite) component of a binary liquid mixture as (than) the microdisk. These areas depend on the lateral position of the microdisk (*i.e.*, x and y) and on the pattern on the substrate. Accordingly, this part of the calculation is purely geometric; we note that the areas satisfy the relation $A_{\mathrm{same}} + A_{\mathrm{opposite}} = \pi R^2$.

In order to further simplify the problem, we neglect the motion of particle in the direction perpendicular to the plane, and assume that it is always located at $z = z_0(x,y)$, which is the position where the potential $U_{\parallel}$ has a minimum with respect to z and, therefore, the probability of finding the particle at $z = z_0$ is biggest. The effective, two-dimensional potential in which the microdisk is moving is given by

$$U_{\parallel}^{\min}(x,y) = U_{\parallel}(x,y,z_0), \quad \text{(S31)}$$

where $z_0 > W/2$, and for $z = z_0$ we have $\partial U_{\parallel}/\partial z = 0$ and $\partial^2 U_{\parallel}/\partial z^2 > 0$.

We calculated the interaction energy $U_{\parallel}^{\min}(x,y)$ numerically using a specially developed computer program written in C++. For $R/\xi \simeq \Gamma = 13.83$ (which corresponds to the temperature

closest to the critical temperature achieved experimentally), this interaction energy is shown in Fig. 5c in the main text.

2. Disk trajectory

In order to calculate the microdisk trajectory, we assumed an overdamped dynamics, which is justifiable for micro-sized disks. Moreover, we neglected Brownian motion, which implies that we consider only a mean trajectory and ignore the microdisk thermal fluctuations around this mean path. These assumptions imply that the force stemming from the potential counterbalances the friction force. Considering the case that the microdisk moves along the curve defined by $x = 0$ and $z = z_0(x = 0, y)$, the corresponding equation of motion reads

$$-\frac{\partial U_{\parallel}^{\min}}{\partial y} = \gamma_{\mathrm{f}} \frac{\mathrm{d}\, y}{\mathrm{d}\, t}. \tag{S32}$$

The friction coefficient γ_{f} depends on $z_0(x = 0, y)$. This dependence can be estimated from the diffusion constant $\mathcal{D}$ using the Sutherland-Einstein-Smoluchowski relation

$$\mathcal{D} = k_{\mathrm{B}} T / \gamma_{\mathrm{f}} \tag{S33}$$

and the experimental data for $\mathcal{D}$. In order to simplify our estimate of $\mathcal{D}$, we make several assumptions. In line with our model (and in agreement with experimental observations), we consider microdisks remaining parallel to the substrate at all times. Additionally, we assume that the lateral diffusion of the microdisk is solely influenced by the solvent hydrodynamics, while the surface patterning affects the diffusion coefficient $\mathcal{D}$ only by altering the microdisk position z_0 above the substrate in order to minimise the substrate–microdisk interaction potential U. Since typical values of z_0 minimising U are much larger than the correlation length (*e.g.*, for the smallest value $\Delta T \approx -0.02\,\mathrm{K}$ accessible experimentally and for taking $z_0/R = 0.25$, one finds $z_0/\xi \approx 3.5$), we neglect the variation of the local composition of the water-2,6-lutidine mixture between the microdisk

and the substrate. Furthermore, we disregard the temperature dependence of $\mathcal{D}$ itself, which is justified given the minute changes in temperature in our experiments. With these assumptions, we utilize our experimental data for the circular pattern (Fig. 2a in the main text) in order to estimate $\mathcal{D}$. In Fig. S11, we plot the experimentally determined diffusion constant as a function of z_0. We employed the least squares method and found a linear relation between $\mathcal{D}$ and z_0 (defined in Eq. (S31)):

$$\mathcal{D} \approx 2.02\,\frac{z_0}{R} - 0.5, \quad \text{(in units of µm}^2\text{/s)}. \tag{S34}$$

In order to calculate the microdisk trajectories, we solved the equation of motion (Eq. (S32) with Eqs. (S33) and (S34)) numerically. We split the whole distance from the bottom to the tip of a triangle into slices of the width $\Delta y = 0.001\,\mu\text{m}$ and estimated the time needed for a microdisk to travel this distance as

$$t_i = t_{i-1} - \frac{k_\mathrm{B}T}{\mathcal{D}(z_0(y_i))}(y_i - y_{i-1})\left(\frac{\partial U_\parallel^\mathrm{min}}{\partial y}\right)^{-1}, \tag{S35}$$

which we derived by replacing the derivative $\mathrm{d}\,y/\,\mathrm{d}\,t$ in Eq. (S32) by its finite difference ratio $(y_i - y_{i-1})/(t_i - t_{i-1})$. In the above equation, we used the value of $\mathcal{D}$ calculated according to Eq. (S34) at z_0 determined by Eq. (S31). We computed the derivative of $U_\parallel^\mathrm{min}$ in Eq. (S35) numerically by calculating the potential at two points separated by $10^{-6}\,\mu\text{m}$. In all calculations we took, as before, $R/\xi \simeq \Gamma = 13.83$. The result is shown in Fig. 5d.

F. Interaction potential between a microsphere and a patterned substrate

The potential of interaction between a spherical particle and a substrate with a circular pattern, as presented in Fig. 1(g) of the main text, has been calculated in the same way as for the microdisk (see section S1 B).

We denote the radius of a microsphere by R and describe the position of the microsphere by the distance D_c between the center of the microsphere and the substrate as well as by a lateral

shift s, which is the distance between the center of the pattern and the orthogonal projection of the center of the microsphere onto the substrate. We note that, in this case, there are no angular degrees of freedom. We assume that the radius R, the mass density ϱ_{p}, the surface charge density σ, and the Debye length λ_{D} are the same as in the case of the microdisk (see table S1).

Following Eq. (S5), we calculate the total potential of interaction U^{sphere} as the sum of the gravitational term $U_{\mathrm{g}}^{\mathrm{sphere}}$, the critical Casimir term $U_{\mathrm{c}}^{\mathrm{sphere}}$, and the electrostatic term $U_{\mathrm{e}}^{\mathrm{sphere}}$. The gravitational potential can be expressed straightforwardly as

$$U_{\mathrm{g}}^{\mathrm{sphere}} = \frac{4}{3}\pi R^4 \Delta_{\mathrm{c}} \left(\varrho_{\mathrm{p}} - \varrho_{\mathrm{l}}\right) g, \tag{S36}$$

where $\Delta_{\mathrm{c}} = D_{\mathrm{c}}/R$ is the reduced height. Similarly as for the microdisk, we have calculated the critical Casimir and the electrostatic potential by using the Derjaguin approximation (see Eq. (S7)). We note that the local distance $\ell\,(x, y)$ between the particle and the substrate, as present in Eq. (S7), encodes the shape of the particle and, therefore, is different for the microdisk and for the microsphere.

The potentials $U_{\mathrm{c}}^{\mathrm{sphere}}$ and $U_{\mathrm{e}}^{\mathrm{sphere}}$ were calculated numerically using a specially written C++ program. The results for the total potential are presented in Fig. 1(g).

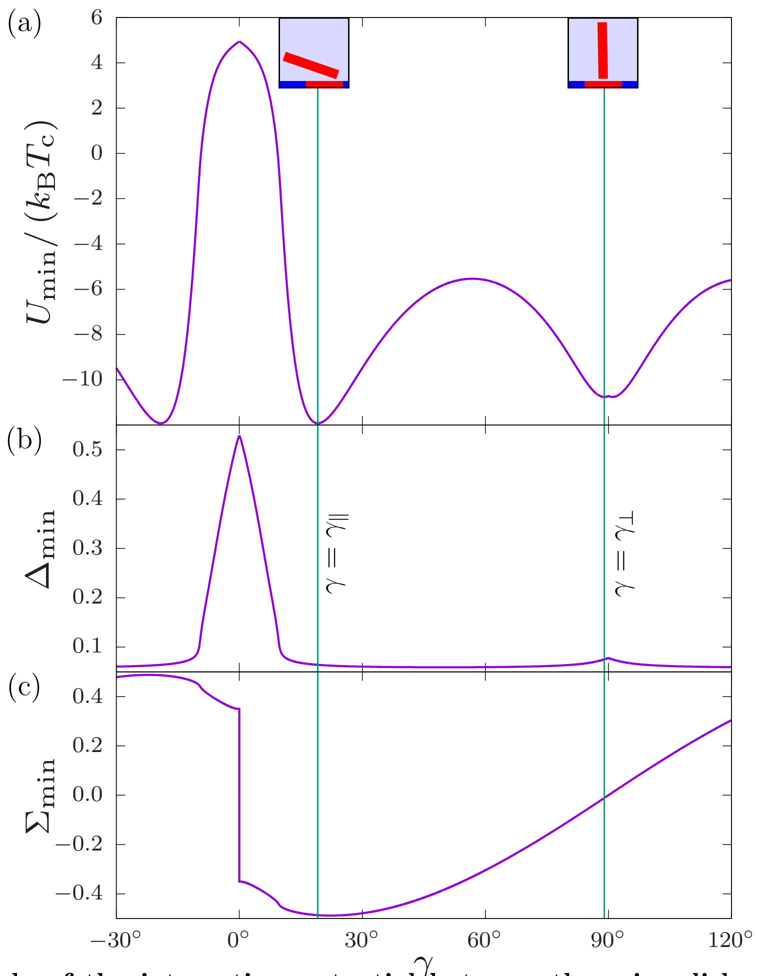

Figure S9. **Example of the interaction potential between the microdisk and the substrate.** Configurations of the microdisk which minimise the total energy for $a/R = \rho = 0.65$ and $R/\xi \simeq \Gamma = 10$ as a function of the tilt angle γ. (**a**) Potential minimum $U_{\rm min}$ as function of γ. The potential is minimised with respect to Δ and Σ for $\alpha = 0°$ and for fixed γ. It attains its minima at $\gamma = \gamma_\parallel$ and $\gamma = \gamma_\perp$. (**b**) $\Delta_{\rm min}$ is the reduced surface-to-surface distance for the configuration which minimises the potential $U_{\rm min}$. (**c**) $\Sigma_{\rm min}$ is the reduced lateral shift of the microdisk for the configuration which minimises $U_{\rm min}$. The green vertical lines represent two configurations for which the potential exhibits local minima with respect to all configurational degrees of freedom. They correspond to two possible configurations with parallel and perpendicular orientations, respectively. The insets in panel (a) show schematic plots of these two configurations. Source data are provided as a Source Data file.

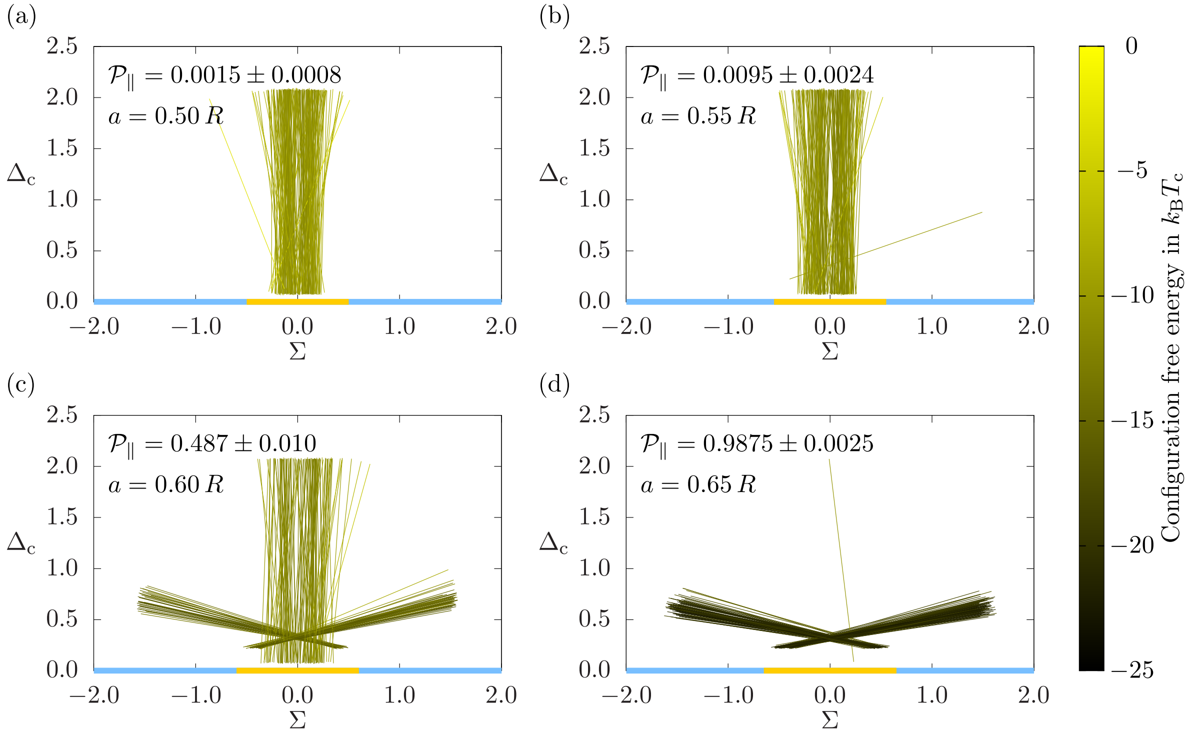

Figure S10. **Illustration of Monte Carlo results for microdisk orientations.** Configurations of 200 non-interacting microdisks obtained by Monte Carlo simulations for $\Gamma = 19 \simeq R/\xi$ and for pattern sizes (**a**) $a = 0.50R$, (**b**) $a = 0.55R$, (**c**) $a = 0.60R$, and (**d**) $a = 0.65R$. Each colored line in the graphs represents a final configuration of a microdisk with the center of the segment located at the same point as the center of the microdisk. The orientation of each microdisk is illustrated by the tilts of the colored lines (segments) by the angle ϕ, such that the angle between the substrate and the circular sides of the microdisk is equal to the angle between the segment and the horizontal axis (see the main text). The color denotes the energy of the configuration in accordance with the color bar. The pattern on the substrate is marked with the blue and orange bars on top of the horizontal axes. As presented in Fig. 2(c), for small pattern sizes a, almost all microdisks are in a perpendicular configuration. Upon increasing a, the parallel configuration starts to dominate. The corresponding probabilities for the parallel configuration are indicated in the left top corner of each panel. Their values, together with uncertainties, have been estimated based on the results of 10 independent runs. Source data are provided as a Source Data file.

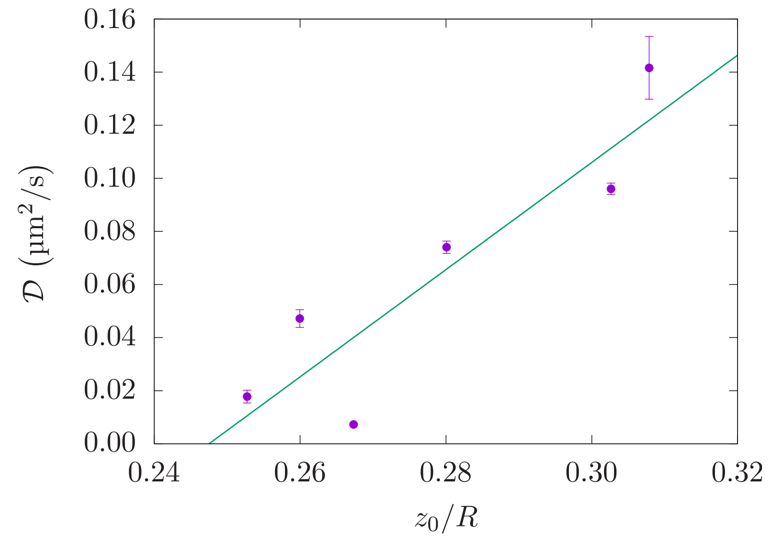

Figure S11. **Dependence of the microdisk diffusion constant on its distance from the substrate.** Dependence of the diffusion constant $\mathcal{D}$ of the microdisk particle in parallel configuration on the distance z_0 between its center and the substrate. The data points have been measured for a microdisk above a circular pattern for various temperatures (see Fig. 2(a) in the main text). The line presents the linear fit given by Eq. (S34). Source data are provided as a Source Data file.

S2. SUPPLEMENTARY REFERENCES

[1] P. H. Jones, O. M. Maragò, and G. Volpe, *Optical Tweezers: Principles and Applications* (Cambridge University Press, Cambridge, 2015).

[2] B. Midtvedt, S. Helgadottir, A. Argun, J. Pineda, D. Midtvedt, and G. Volpe, Quantitative digital microscopy with deep learning, Appl. Phys. Rev. **8**, 011310 (2021).

[3] S. Helgadottir, A. Argun, and G. Volpe, Digital video microscopy enhanced by deep learning, Optica **6**, 506 (2019).

[4] B. Midtvedt, J. Pineda, F. Skärberg, E. Olsén, H. Bachimanchi, E. Wesén, E. K. Esbjörner, E. Selander, F. Höök, D. Midtvedt, and G. Volpe, Single-shot self-supervised object detection in microscopy, Nat. Commun. **13**, 7492 (2022).

[5] F. Schmidt, A. Callegari, A. Daddi-Moussa-Ider, B. Munkhbat, R. Verre, T. Shegai, M. Käll, H. Löwen, A. Gambassi, and G. Volpe, Tunable critical Casimir forces counteract Casimir–Lifshitz attraction, Nat. Phys. **19**, 271 (2023).

[6] S. K. Lamoreaux, Demonstration of the Casimir force in the 0.6 to 6 μ m range, Phys. Rev. Lett. **78**, 5 (1997).

[7] U. Mohideen and A. Roy, Precision measurement of the Casimir force from 0.1 to 0.9 μ m, Phys. Rev. Lett. **81**, 4549 (1998).

[8] A. Le Cunuder, A. Petrosyan, G. Palasantzas, V. Svetovoy, and S. Ciliberto, Measurement of the Casimir force in a gas and in a liquid, Phys. Rev. B **98**, 201408 (2018).

[9] Archimedes of Syracuse, On floating bodies. Book I., in *The works of Archimedes*, edited by T. L. Heath (University Press, Cambridge, 1897) p. 253.

[10] C. Hertlein, L. Helden, A. Gambassi, S. Dietrich, and C. Bechinger, Direct measurement of critical Casimir forces, Nature **451**, 172 (2008).

[11] O. Vasilyev, A. Gambassi, A. Maciołek, and S. Dietrich, Universal scaling functions of critical Casimir forces obtained by Monte Carlo simulations, Phys. Rev. E **79**, 041142 (2009).

[12] N. Farahmand Bafi, P. Nowakowski, and S. Dietrich, Effective pair interaction of patchy particles in critical fluids, J. Chem. Phys. **152**, 114902 (2020).

[13] F. Schmidt, A. Callegari, A. Daddi-Moussa-Ider, B. Munkhbat, R. Verre, T. Shegai, M. Käll, H. Löwen, A. Gambassi, and G. Volpe, Tunable critical casimir forces counteract casimir–lifshitz attraction, Nature Physics **19**, 271 (2022).

[14] V. A. Parsegian, *Van der Waals Forces* (Cambridge University Press, New York, 2006).

[15] A. Gambassi, A. Maciołek, C. Hertlein, U. Nellen, L. Helden, C. Bechinger, and S. Dietrich, Critical Casimir effect in classical binary liquid mixtures, Phys. Rev. E **80**, 061143 (2009).

[16] M. Galassi and B. Gough, *GNU Scientific Library: Reference Manual*, GNU manual (Network Theory Limited, Bristol, 2009).

[17] N. Metropolis, A. W. Rosenbluth, M. N. Rosenbluth, A. H. Teller, and E. Teller, Equation of state calculations by fast computing machines, J. Chem. Phys. **21**, 1087 (1953).
