# Supplementary material for: Nanoalignment by critical Casimir torques
Source: Nat Commun. 2024 Jun 14;15:5086. doi: 10.1038/s41467-024-49220-1 (PMC11178905; doi:10.1038/s41467-024-49220-1)
Supplement: Supplementary file 3 — Description of Additional Supplementary Files [file 41467_2024_49220_MOESM3_ESM.docx]

**File Name: Supplementary Video 1**

**Description:** Bright-field videos of the movement of 3 μm diameter microspheres and 2.4 μm diameter microdisks above a 2.2 μm diameter circular pattern at ∆T ≈ −0.30 K off the critical temperature T_c_ and T ≈ T_c_. The red and blue crosses represent the tracked particle center points. The cyan dotted circles depict the pattern on the substrate, while the white dotted circles outline the particles.

**File Name: Supplementary Video 2**

**Description:** Videos captured in bright-field microscopy showing the motion of 2.4 μm diameter microdisks above a 2.2 μm diameter circular pattern at ∆T ≈ −0.70 K, ∆T ≈ −0.50 K, ∆T ≈ −0.10 K off the critical temperature T_c_. The red crosses represent the tracked particle center points. The cyan dotted circles depict the pattern on the substrate, while the white dotted circles outline the particles.

**File Name: Supplementary Video 3**

**Description:** Videos captured in bright-field microscopy showing the motion of 2.4 μm diameter microdisks above a 2.2 μm diameter circular pattern and elliptical pattern (long axis 2.2 μm and short axis 0.6 μm) at T ≈ T_c_. The red crosses represent the tracked particle center points. The cyan dotted circles depict the pattern on the substrate, while the white dotted circles outline the particles.

**File Name: Supplementary Video 4**

**Description:** Videos captured in bright-field microscopy showing the motion of chiral microparticles. Each microparticle is composed of two conjoined, partially overlapping rectangles, each with a length of 2.8 μm and a height of 1.8 μm. The microparticles hover above chiral patterns with the same shape (slightly smaller, with a length of 2.5 μm and a width of 1.7 μm) at T ≈ T_c_. The blue crosses represent the tracked particle center points. The white dashed boxes outline the particles.

**File Name: Supplementary Video 5**

**Description:** Videos captured in bright-field microscopy showing the motion of 2.4 μm diameter microdisks above triangular patterns with a 2 μm base and different heights (18 μm, 26 μm, 30 μm, and 36 μm) at T ≈ T_c_. The white dotted circles outline the microdisks, while the red lines represent the tracked trajectories. The video has been accelerated five times.

**File Name: Supplementary Video 6**

**Description:** Top: Videos captured in bright-field microscopy showing the motion of a 2.4 μm diameter microdisk above a pattern constituted by a series of trapezoids with a height of 18 μm and short and wide bases of 1 μm and 2 μm. The white dotted circles outline the microdisk, while the red line represents the tracked trajectory over the last 40 s. Below: Temperature difference ∆T = T − T_c_ varies throughout the entire video, while the red marker represents the ∆T corresponding to the current frame. The video has been accelerated twenty-five times.

**File Name: Supplementary Video 7**

**Description:** Left: Videos captured in bright-field microscopy showing the motion of 2.4 μm diameter microdisk above a curved trapezoidal bull-eye pattern. The white dotted circles outline the microdisk, while the red line represents the tracked flake trajectory over the last 25 s. Right: Temperature difference ∆T = T − T_c_ varies throughout the entire video, while the red marker represents the ∆T corresponding to the current frame. The video has been accelerated five times.
