# Supplementary figures and images for: Nanoalignment by critical Casimir torques
Source: Nat Commun. 2024 Jun 14;15:5086. doi: 10.1038/s41467-024-49220-1 (PMC11178905; doi:10.1038/s41467-024-49220-1)

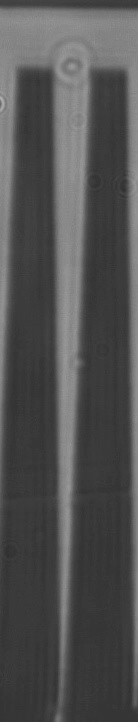

Supplement: Supplementary file 11 — Source Data [file 41467_2024_49220_MOESM11_ESM.zip › fig5/python/img/Background.jpg]

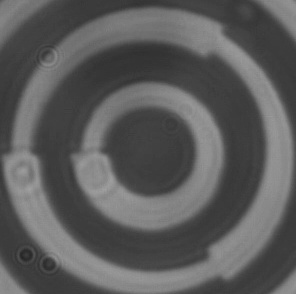

Supplement: Supplementary file 11 — Source Data [file 41467_2024_49220_MOESM11_ESM.zip › fig5/python/img/Circle.jpg]

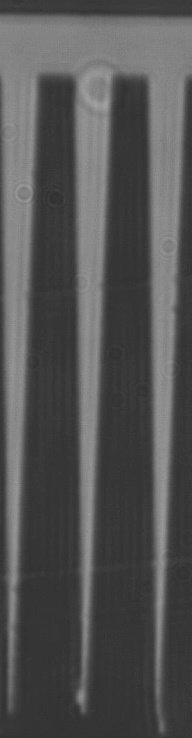

Supplement: Supplementary file 11 — Source Data [file 41467_2024_49220_MOESM11_ESM.zip › fig5/python/img/Triangle.jpg]

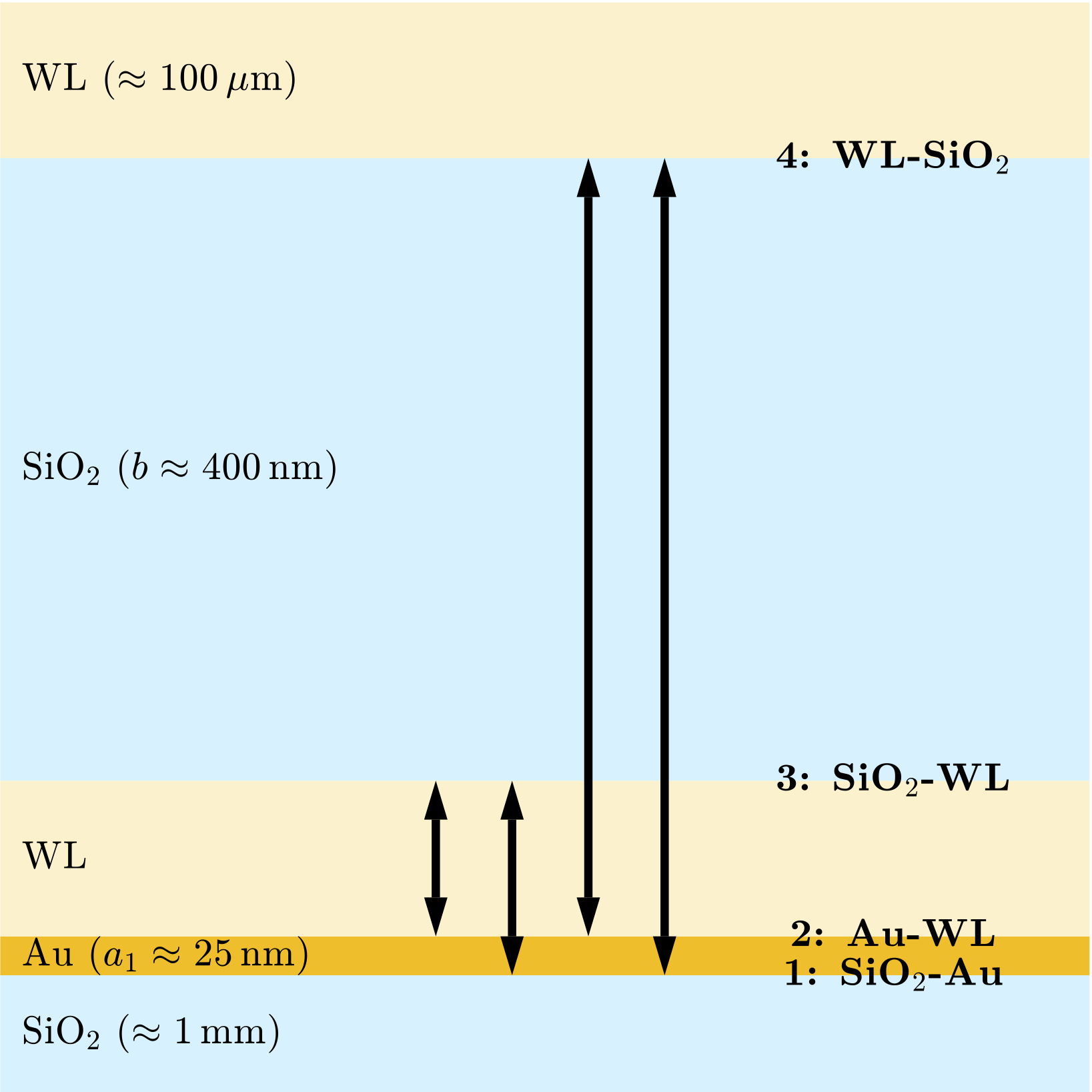

Supplement: Supplementary file 11 — Source Data [file 41467_2024_49220_MOESM11_ESM.zip › figS7/glass_on_coated_glass.png]

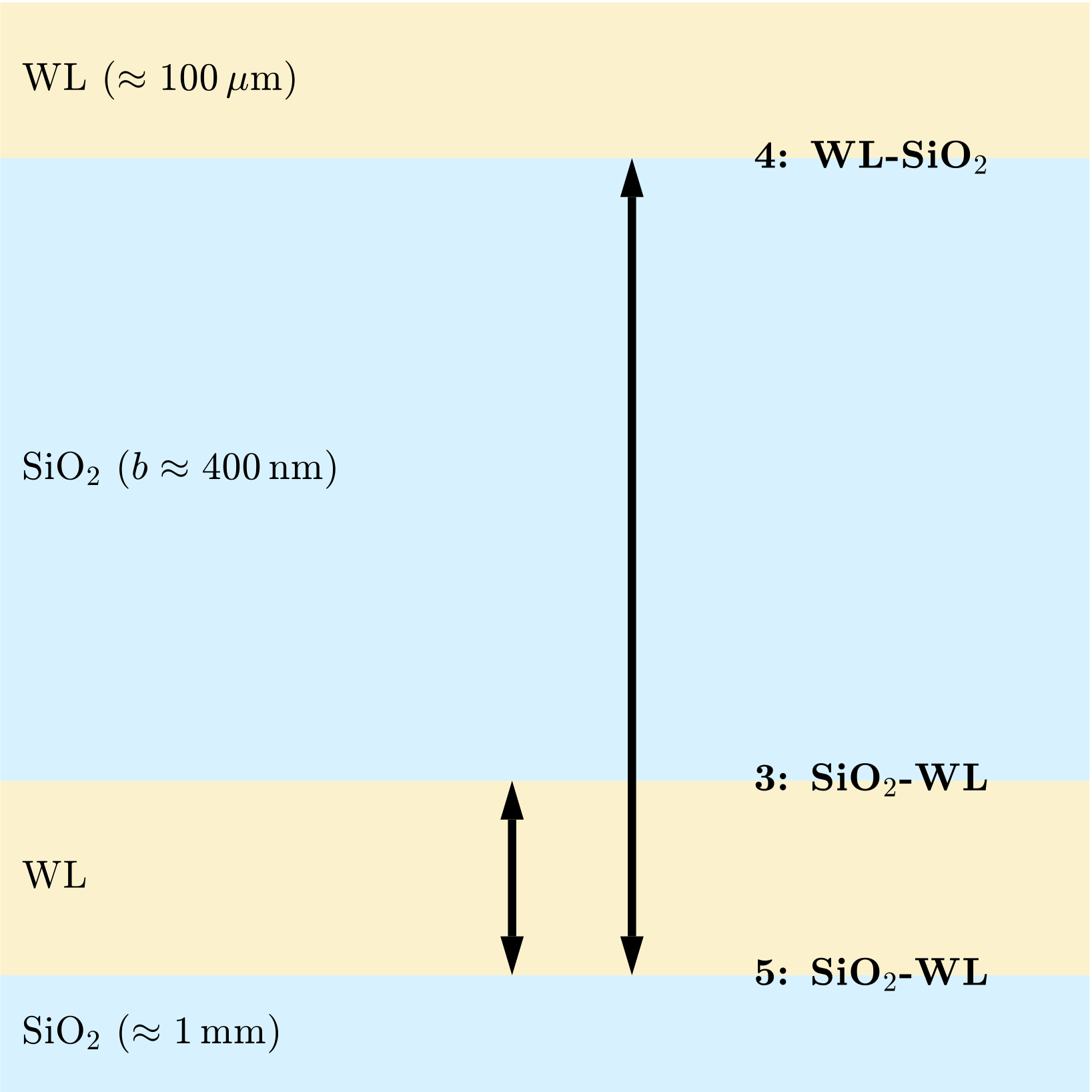

Supplement: Supplementary file 11 — Source Data [file 41467_2024_49220_MOESM11_ESM.zip › figS7/glass_on_glass.png]
